# CircZFR functions in cancer from molecular networks to precision therapy

**DOI:** 10.3389/fgene.2025.1694220

**Published:** 2025-10-14

**Authors:** Jinniang Nan, Min Li, Xinru Li, Yuqing Guo, Ziye Cai, Zekang Deng, Chengyi Zhan, Jiacheng Li, Shuying Liu, Ziyi Chen, Zhenjun Huang, Yihan Yang

**Affiliations:** ^1^ School of Clinical Medicine, Nanchang Medical College, Nanchang, Jiangxi, China; ^2^ Zhongshan School of Medicine, Sun Yat-sen University, Guangzhou, China; ^3^ Department of Medical Oncology, Sun Yat-sen Memorial Hospital, Sun Yat-sen University, Guangzhou, Guangdong, China; ^4^ Jiangxi Provincial Key Laboratory of Respiratory Diseases, Jiangxi Institute of Respiratory Diseases, The Department of Respiratory and Critical Care Medicine, The First Affiliated Hospital, Jiangxi Medical College, Nanchang University, Nanchang, China

**Keywords:** circRNA, circZFR, cancer, oncogenic signaling, tumor progression

## Abstract

CircRNAs are a class of covalently closed circular non-coding RNAs formed through back-splicing of precursor mRNAs, lacking 5′ caps and 3′ poly(A) tails, and are widely present in eukaryotic cells. Advances in high-throughput sequencing have revealed that circRNAs regulate gene expression and participate in disease pathogenesis, often exhibiting dual oncogenic or tumor-suppressive roles in cancer. CircZFR primarily comprises two subtypes—hsa_circ_0072088 and hsa_circ_0000345—which are derived from exons of the ZFR gene located on chromosome 5q13.3. CircZFR plays a critical regulatory role in the progression of multiple malignancies. This review systematically summarizes recent research progress on circZFR across different cancer types, elucidating its molecular mechanisms in influencing tumor proliferation, invasion, metastasis, and drug resistance through acting as a miRNA sponge, modulating signaling pathways, or directly binding to proteins. Furthermore, this review integrates the interaction networks between circZFR and key regulatory factors, unveiling its multifaceted functions in remodeling the tumor microenvironment. This review emphasizes the aberrant expression patterns of circZFR and their clinical prognostic relevance in multiple cancers, including colorectal, pancreatic, thyroid, bladder, hepatocellular, breast, lung, and cervical carcinomas, discusses its potential as a novel diagnostic biomarker or therapeutic target, and aims to provide a theoretical basis for precision cancer therapy while highlighting its translational research prospects.

## Introduction

Circular RNA (CircRNA) is a class of non-coding molecules made of single-stranded RNA that forms a closed-loop structure through circularization, with covalent linkage between the 3′ and 5′ ends. Because CircRNA lacks a 5′ cap structure and a 3′ poly(A) tail, it is highly stable and resistant to nuclease degradation. Furthermore, most CircRNAs show significant evolutionary conservation across species (Greene et al., 2017). CircRNAs can consist of introns, exons, or both, and are widely present in various organisms, showing notable evolutionary conservation (Wang et al., 2019). Initially, CircRNA was thought to be a byproduct of splicing errors. However, with advancements in RNA sequencing technology, it has been reevaluated, and studies show that it stably exists in healthy tissues (Wang et al., 2019). Currently, CircRNA research mainly focuses on humans and model organisms, particularly its potential functions and mechanisms in diseases like cancer (Yalamarty et al., 2023).

CircRNAs play key roles in cancer onset and progression, regulating tumor cell proliferation, migration, invasion, immune evasion, and tumor microenvironment (TME) remodeling (Seguella and Gulbransen, 2021; Wu et al., 2021; Yan and Chen, 2020). Initially, CircRNAs were believed to regulate gene expression by acting as “sponges” for microRNAs (miRNAs), preventing their binding to target genesAs research has advanced, it has been found that CircRNAs not only act as “sponges” for miRNAs but also serve as “sponges” or decoys for proteins, recruiting them, enhancing their functions, or acting as protein scaffolds, thereby regulating cellular functions at multiple levels (Chen, 2020; Kristensen et al., 2019). CircRNAs often act synergistically through multiple mechanisms to regulate tumor-related signaling pathways, thereby promoting cancer initiation, progression, and metastasis (Chen et al., 2024; Wang et al., 2023; Xiong et al., 2021). As key regulatory molecules, circRNAs have become a critical focus in tumor mechanism research and targeted therapy development.

Circular RNA zinc finger RNA-binding protein (CircZFR) is the transcriptional product of the zinc finger RNA-binding protein (ZFR) gene, located on human chromosome 5p13.3. CircZFR plays a dual role in multiple human cancers, acting both as a tumor suppressor and a tumor promoter (Li et al., 2021). Recent studies have shown that CircZFR is closely associated with disease progression in various cancers, including hepatocellular carcinoma (HCC) (Luo et al., 2020; Tan et al., 2019; Xu et al., 2021; Yang et al., 2019), papillary thyroid carcinoma (PTC) (Wei et al., 2018), lung cancer (LC) (Li et al., 2020; Ma et al., 2023; Ren et al., 2020; H. Zhang et al., 2019), bladder cancer (BLCA) (Luo et al., 2020; W.-Y. Zhang et al., 2019), breast cancer (BC) (Chen et al., 2020), cervical cancer (CCA) (Zhou et al., 2021), pancreatic cancer (PC) (Wang et al., 2023), thyroid cancer (TC) (Wei et al., 2018; Xiong et al., 2021), and colorectal cancer (CRC) (Bian et al., 2018; Chen et al., 2024; Tan et al., 2022). Therefore, investigating the functions of circZFR in human cancers and its clinical potential is of great importance (Zhang et al., 2025). This paper will conduct a comprehensive analysis of the functions of CircZFR in various types of cancer, aiming to explore its potential for clinical application in depth.

### The sponge-like role of CircZFR in tumorigenesis

CircZFR primarily functions as a molecular sponge in tumor progression, sequestering target miRNAs to modulate downstream signaling and promote tumor development (Wang et al., 2023; Yang et al., 2019). Among the miRNAs regulated by CircZFR, miR-357 has been identified as a potential biomarker in pancreatic and liver cancer cells. CircZFR functions as a molecular “sponge” for miR-357, thereby promoting tumor cell proliferation, metastasis, and invasion (Bloomston et al., 2007; Li et al., 2024). Additionally, CircZFR is associated with miR-532-3p, which promotes apoptosis and inhibits cell cycle progression in CRC cells by regulating the transcription of downstream FOXO4; However, CircZFR also promotes CRC cell proliferation and metastasis by modulating the miR-3127-5p/RTKN2 and miR-147a/CACUL1 axes (Chen et al., 2024; Du et al., 2017; Tan et al., 2022). Through these two distinct regulatory mechanisms, CircZFR can exert opposing effects, either promoting or inhibiting tumor progression. These opposing mechanisms underline its context-dependent role in tumor progression (Kristensen et al., 2022; Wang et al., 2023). CircZFR drives BLCA progression by orchestrating a multi-axis regulatory network, including the miR-3619-5p/CTNNB1, miR-511/AKT1, STAT3/NF-κB, and miR-624-3p/WEE1 pathways (Czauderna et al., 2019; Tan et al., 2019; Yang et al., 2019; Zhang et al., 2024). Additionally, CircZFR promotes glycolysis and tumor cell colony formation by acting as a sponge for miR-375 and selectively splicing MYO1B via AKT-mTOR signaling to enhance OXPHOS. It further influences the miR-16/MAPK1, miR-545/1270/Wnt5a, miR-377/ZEB2, miR-578/HIF1A, and SSBP1/CDK2/cyclin E1 axes in thyroid, bladder, breast, and cervical cancers, respectively (Yu et al., 2019).

### Molecular characteristics and clinical implications of CircZFR in cancer

**FIGURE 1:**
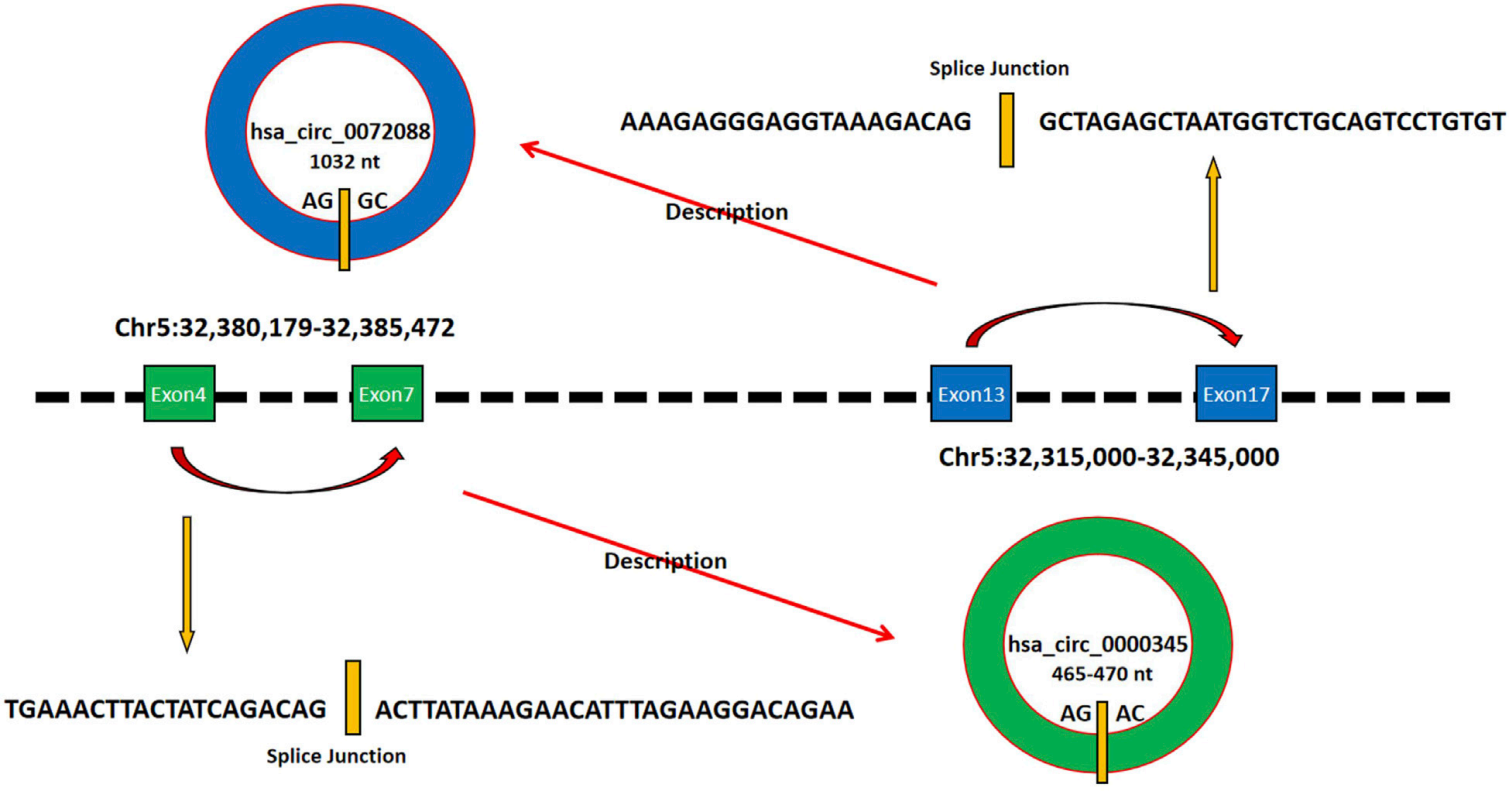
Exons 4 to 7 of the hsa_circ_0000345 gene and exons 13 to 17 of the hsa_circ_0072088 gene are both located on chromosome 2, which can form hsa_circ_0000345 with 465-470 nt and hsa_circ_0072088 with 1032 nt, respectively, as shown in the schematic diagram.

CircZFR is a circular RNA formed by reverse splicing of the pre-mRNA from its host gene ZFR, which is located on human chromosome 5p15.33 (Wei et al., 2018). Currently reported circZFR primarily consists of two subtypes—hsa_circ_0000345 and hsa_circ_0072088 (most studies focus on hsa_circ_0072088). The circularization of hsa_circ_0000345 relies on reverse splicing between the 3′ splice site of exon 4 and the 5′ splice site of exon 7 in the ZFR pre-mRNA, forming a closed circular structure encompassing exons 4–7 (Wasinska-Kalwa et al., 2025). Its length ranges approximately from 465 to 470 nt, depending on the precise cleavage of splicing sites during circularization (Zhang et al., 2016). hsa_xirc_0072088 is a circular RNA formed by reverse splicing of exons 13–17 of the ZFR gene, with a length of 1,032 nt (Chen et al., 2024). Both subtypes lack a 5′ cap and 3′ polyA tail. Their closed-loop structure confers high stability and exonuclease resistance, prolonging their intracellular half-life (Wei et al., 2018). circZFR is primarily localized in the cytoplasm (Ma et al., 2023). Clinical cohort analysis revealed that its high expression is significantly associated with reduced overall survival in patients (Bongolo et al., 2025). This suggests that circZFR may serve not only as a potential tool for clinical diagnosis and prognostic assessment but also as a therapeutic intervention target worthy of further exploration. The details of its molecular characteristics are summarized in Figure 1.

### CircZFR expression and its roles in cancer

**FIGURE 2:**
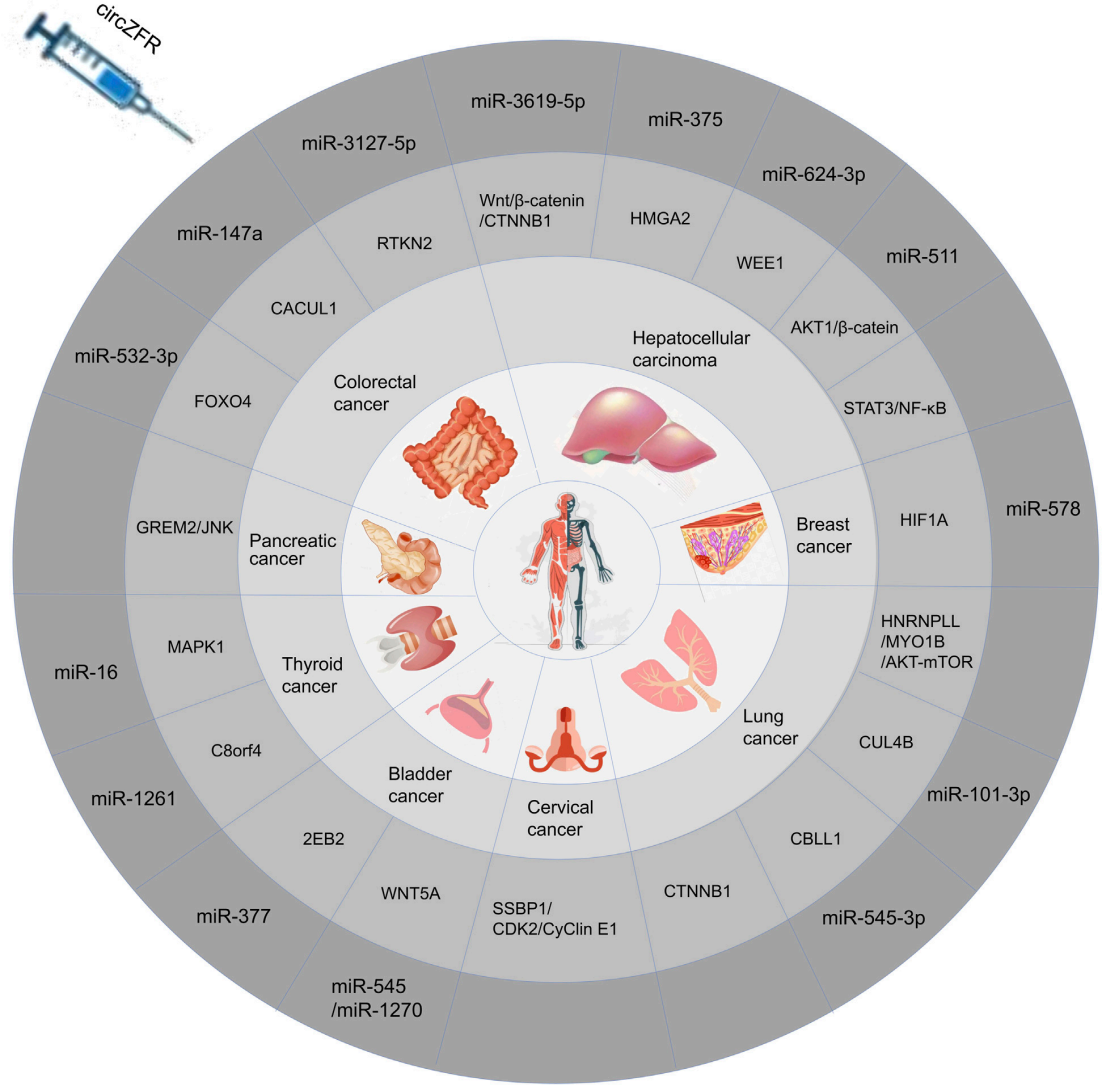
CircZFR participates in various diseases through different ways.

With the advancement of circRNA research, CircZFR has been demonstrated to be abnormally expressed in multiple cancers, including colorectal cancer (Chen et al., 2024), pancreatic cancer (Wang et al., 2023), thyroid cancer (Wei et al., 2018), bladder cancer (W.-Y. Zhang et al., 2019), hepatocellular carcinoma (Xu et al., 2021), breast cancer (Chen et al., 2020), and lung cancer (Ren et al., 2020). CircZFR plays an oncogenic role in the vast majority of cancer types by regulating multiple signaling pathways through miRNA trapping or direct binding to signaling molecules. This promotes tumor cell proliferation, migration, invasion, and resistance to apoptosis. Furthermore, CircZFR expression levels correlate significantly with tumor malignancy and poor prognosis, suggesting its potential as a diagnostic biomarker and therapeutic target. However, in certain cancers, CircZFR knockout inhibits cell cycle progression and induces apoptosis, suggesting its expression level is closely linked to tumor malignant phenotypes. Furthermore, CircZFR participates in regulating tumor drug resistance, mediating cisplatin resistance in HCC and LC. The expression of CircZFR undergoes dynamic regulation during tumor progression, with its overexpression significantly associated with poor prognosis (Zhou et al., 2022). This not only positions it as a potential diagnostic biomarker but also provides crucial evidence for developing novel targeted therapeutic strategies. Furthermore, CircZFR participates in regulating tumor drug resistance, mediating resistance to cisplatin in HCC and LC. The details of its diverse roles are summarized in Table 1 and Figure 2.

**TABLE 1:** CircZFR in tumor progression.

Cancer type	Level	Biological function	Related miRNA genes	Target axis	References
Colorectal cancer	Up	Promoted cell growth, spreading, proliferation and migration Inhibited apoptosis	miR-3127-5p	CircZFR-miR-3127-5p/RTKN2	PMID:38805063
	Up	Promoted apoptosis Inhibited proliferation and cell cycle progression	miR-147a	CircZFR-miR-147a/CACUL1	PMID:36092343
	Down	Enhanced cell proliferation, migratory and apoptosis Inhibited cell cycle progression	miR-532-3p	CircZFR- miR-532-3p/FOXO4	PMID:30249393
Pancreatic cancer	Up	Promoted proliferation and migration Enhanced tumorigenicity	miR-375	CircZFR- miR-375/GREM2/JNK	PMID:36990335
Thyroid cancer	Up	Promoted cell cycle progression, cell growth and migratory Inhibited apoptosis and proliferation	miR-16	CircZFR- miR-16/MAPK1	PMID:34323000
	Up	Promoted proliferation, migratory and invasion	miR-1261	CircZFR- miR-1261/C8orf4	PMID:29842886
Bladder cancer	Up	Promoted cell growth, migration, invasion and cell cycle progression Inhibited apoptosis	miR-377	CircZFR- miR-377/ZEB2	PMID:31746333
	Up	Promoted proliferation, migration and invasion	miR-545, miR-1270	Circ-ZFR miR-545, CircZFR-miR-1270	PMID:33928018
Hepatocellular carcinoma	Up	Promoted proliferation	miR-3619-5p	CircZFR- miR-3619-5p/CTNNB1	PMID:30468709
	Up	Promoted proliferation, glycolysis and cell growth Inhibited apoptosis	miR-375	CircZFR- miR-375/HMGA2	PMID:33433801
	Up	Promoted proliferation, migration and invasion Inhibited apoptosis	miR-511	CircZFR- miR-511/AKT1	PMID:31147216
	Up	Promoted cell growth Enhanced resistance of the HCC cells	NA	CircZFR-STAT3/NF-κB	PMID:35139763
	Up	Promoted proliferation, migration and invasion Inhibited apoptosis	miR-624-3p	CircZFR- miR-624-3p/WEE1	PMID:37516591
Breast cancer	Up	Promoted cell viability, colony formation, migration, invasion, cell growth and glycolysis Inhibited apoptosis	miR-578	CircZFR- miR-578	PMID:32831653
Lung cancer	Up	Promoted cell growth, proliferation and cell cycle progression	miR-326	CircZFR-miR-326	PMID:35117697
	Up	Promoted proliferation, migration and invasion	miR-101-3p	CircZFR- miR-101-3p	PMID:31407591
	Up	Promoted apoptosis Inhibited colony formation, migration and cell cycle progression	miR-545-3p	CircZFR- miR-545-3p/CBLL1	PMID:32190002
	Up	Enhanced oxidative phosphorylation	NA	CircZFR-HNRNPLL/MYO1B/AKT-mTOR	PMID:37461053
Cervical cancer	Up	Promoted proliferation, invasion and cell growth	NA	CircZFR-SSBP1/CDK2/cyclin E1	PMID:33516252

## CircZFR in colorectal cancer

CRC is one of the most common malignancies worldwide, accounting for 9.4% of all cancer-related deaths. In 2022, CRC accounted for nearly one-tenth of all cancer-related deaths (Morgan et al., 2023).

Recently, CircZFR has been recognized as a potential novel biomarker for the diagnosis, treatment, and prognosis of CRC, exhibiting significant oncogenic activity and promoting CRC progression through various mechanisms. CircZFR directly binds to and stabilizes the BCLAF1 protein, inhibiting its ubiquitination and degradation, thereby promoting CRC cell proliferation and migration. CircZFR also directly regulates cytoskeletal reorganization, migration, and anti-apoptotic processes, and relieves transcriptional repression of RTKN2 by binding to miR-3127-5p. As a key effector molecule in the Rho GTPase signaling pathway, it drives the malignant progression of CRC by inhibiting apoptosis, promoting migration and invasion, and enhancing proliferation, thereby creating a pro-cancer positive feedback loop with CircZFR (Chen et al., 2024). Additionally, CircZFR induces G1/S phase arrest and promotes apoptosis in CRC cells through the miR-147a/CACUL1 axis (Tan et al., 2022). Bian et al. further confirmed that CircZFR dynamically regulates FOXO4 protein expression levels. As a member of the O subclass of the forkhead family of transcription factors, FOXO4 is involved in oxidative stress signaling, proliferation, insulin signaling, apoptosis inhibition, and cell cycle regulation. Knocking out CircZFR reduces FOXO4 expression by 2.3 times.

In summary, CircZFR is a key regulator in CRC progression, driving tumorigenesis through multiple pathways, including stabilizing BCLAF1, relieving miRNA inhibition of the oncogene RTKN2, disrupting the cell cycle, and regulating the transcription factor FOXO4. The core mechanism of action highlights CircZFR’s potential as a therapeutic target, providing key evidence and guidance for further exploration of CRC pathogenesis and the development of new therapies.

## CircZFR in pancreatic cancer

PC is considered one of the most lethal cancers. Although the global incidence is projected to reach 624,000 new cases by 2025—a 40% increase from 2015—the prognosis has remained largely unchanged over recent decades, with a 5-year overall survival rate of only 2%–9% after diagnosis (Salom and Prat, 2022). Effectively addressing this clinical challenge requires a deeper understanding of the complex molecular regulatory mechanisms of PC, as well as the development of novel diagnostic biomarkers and therapeutic targets. CircZFR is highly expressed in PC tissues and cell lines, and its elevated expression correlates negatively with tumor stage, metastasis risk, and overall patient survival. CircZFR promotes PC cell proliferation, migration, and tumorigenicity by activating two key signaling pathways: the BCLAF1-mediated proliferation and anti-apoptotic pathway, and the Rho/RTKN2-mediated cell migration pathway. These findings highlight its potential as an early diagnostic biomarker (Chen et al., 2024). Additionally, CircZFR enhances cell migration and invasion by modulating the expression of proteins involved in EMT. This effect is achieved through specific binding to miR-375, which relieves its inhibition of GREM2, activates the JNK signaling pathway, and consequently promotes cancer cell proliferation, invasion, EMT, and the maintenance of malignant phenotypes. These findings suggest that GREM2-mediated activation of the JNK signaling pathway may represent a conserved mechanism underlying PC progression Wang et al. (2023).

In summary, CircZFR functions as a central oncogenic driver in PC, facilitating tumor progression through the regulation of key signaling pathways, including BCLAF1, Rho/RTKN2, and the miR-375/GREM2/JNK axis. The marked tissue-specific overexpression of CircZFR, along with its strong association with poor prognosis, highlights its potential as an early diagnostic biomarker and therapeutic target in pancreatic PC. A more comprehensive understanding of its regulatory network will offer a crucial theoretical basis for the development of targeted therapies and the improvement of pancreatic cancer’s poor prognosis.

## CircZFR in thyroid cancer

TC is among the most common endocrine malignancies, accounting for approximately 2.8% of all new cancer cases worldwide and showing a continuously increasing incidence (Ferlay et al., 2019). CircZFR has been identified as a critical molecular regulator of the initiation and progression of TC. CircZFR competitively binds to miR-1261 in TC cells, thereby relieving miR-1261–mediated suppression of C8orf4 expression. Restoration of C8orf4 significantly reversed the inhibitory effects of CircZFR knockdown on cell proliferation, migration, and invasion in PTC cells (Wei et al., 2018). Notably, this study further uncovered a bidirectional positive feedback mechanism within this regulatory axis: the C8orf4 protein also functions as a competitive endogenous RNA (ceRNA) for miR-1261, thereby enhancing CircZFR expression and establishing a molecular loop that sustains the malignant phenotype of pancreatic TC cells. Additionally, silencing CircZFR suppresses cell viability and invasion while promoting apoptosis by sponging miR-16 and downregulating MAPK1. CircZFR, which is significantly overexpressed in tTC, exerts its oncogenic function via a ceRNA mechanism. Specifically, CircZFR knockdown increases miR-16 availability by relieving its sponge-like sequestration, thereby restoring miR-16–mediated inhibition of the oncogene MAPK1 and ultimately suppressing TC progression (Xiong et al., 2021).

An in-depth analysis of existing research indicates that while both studies collectively establish the core oncogenic role of CircZFR, they reveal distinct molecular pathways and levels of mechanism. The innovation of Hong et al.'s work lies in breaking through the traditional linear model of ceRNA, first demonstrating the bidirectional positive feedback loop between CircZFR/miR-1261/C8orf4, emphasizing the complexity and self-sustaining nature of molecular regulation. In contrast, Xiong et al.'s work focused on the classic ceRNA axis of CircZFR/miR-16/MAPK1, providing another robust line of evidence for CircZFR’s oncogenic function (Xiong et al., 2021). Notably, neither study fully integrated or discussed the pathways proposed by the other, failing to construct a unified regulatory network. This reflects the current limitation of circRNA functional research often being confined to single molecular axes, while also leaving room for future studies to explore cross-talk and synergistic interactions between different pathways.

In summary, CircZFR serves as a central oncogenic driver in TC, promoting tumor proliferation, migration, invasion, and resistance to apoptosis by establishing a CircZFR/miR-1261/C8orf4 positive feedback loop and modulating the CircZFR/miR-16/MAPK1 signaling axis. Its pivotal role and markedly elevated expression in TC highlight its potential as both a prognostic biomarker for disease progression and a promising therapeutic target.

## CircZFR in bladder cancer

BLCA is among the most common malignancies of the urinary system worldwide. In 2023, BLCA accounted for 4.2% of all newly diagnosed cancer cases and 2.7% of cancer-related deaths, with an overall 5-year relative survival rate of 77.9%. However, enhancing survival outcomes for BLCA remains a significant clinical challenge. Therefore, elucidating the molecular mechanisms underlying its pathogenesis is essential for the discovery of novel therapeutic targets (Leslie et al., 2025).

CircZFR facilitates BLCA progression by modulating multiple signaling pathways. Its knockdown significantly suppresses BLCA cell proliferation, migration, and invasion, while promoting cell cycle arrest and apoptosis. On one hand, CircZFR binds to miR-377, thereby relieving its suppression of the EMT-related transcription factor ZEB2, which in turn promotes EMT and enhances tumor invasiveness. Restoration of ZEB2 expression can reverse the effects of CircZFR downregulation—such as cell cycle arrest and enhanced apoptosis—suggesting that this signaling axis plays a critical role in CircZFR-mediated tumor progression (W.-Y. Zhang et al., 2019). On the other hand, CircZFR also targets miR-545 and miR-1270, thereby relieving their suppression of WNT5A and promoting its upregulation. This, in turn, activates the non-canonical Wnt signaling pathway associated with migration and invasion, ultimately driving tumor cells toward a more invasive phenotype (Luo et al., 2020).

In summary, CircZFR plays a critical role in the initiation and progression of BLCA by modulating two key signaling pathways: miR-377/ZEB2 and miR-545/miR-1270/WNT5A. Its high expression is closely associated with unfavorable clinical features and highlights its potential as both a diagnostic and therapeutic target, underscoring its significant value for research and clinical application.

## CircZFR in hepatocellular carcinoma

HCC ranks as the sixth most common cancer and the third leading cause of cancer-related mortality worldwide, with a 5-year survival rate of merely 18%. Most cases arise in individuals with cirrhosis, with an annual incidence of 2%–4%. According to 2018 data, more than 80% of new HCC cases occur in developing countries with a high prevalence of hepatitis B, and the incidence continues to rise. Current treatment options remain limited, underscoring the urgent need to identify novel molecular targets and develop more effective therapeutic strategies (Asafo-Agyei and Samant, 2025).

CircZFR plays a critical role in the progression of HCC. It is significantly overexpressed in HCC tissues and cell lines and is strongly associated with poor clinical outcomes. Knockdown of CircZFR significantly suppresses HCC cell proliferation, migration, and EMT, while promoting apoptosis, highlighting its pivotal role in HCC progression. CircZFR regulates multiple miRNA–target gene axes to promote the development of malignant phenotypes in HCC. Tan Aichun et al. reported that CircZFR functions as a ceRNA by sponging miR-3619-5p, thereby relieving its suppression of CTNNB1, activating the Wnt/β-catenin signaling pathway, and promoting HCC cell proliferation and EMT (Tan et al., 2019). Additionally, CircZFR functions as a molecular sponge for miR-375, leading to the upregulation of HMGA2. In HCC cells, miR-375 suppresses tumor progression by directly targeting HMGA2, thereby inhibiting cell proliferation, glycolysis, and tumor growth, while promoting apoptosis (Xu et al., 2021). Furthermore, CircZFR binds to miR-511, resulting in the upregulation of AKT1 and activation of key downstream oncogenic proteins, including c-Myc, Cyclin D1, Survivin, and Bcl-2, ultimately accelerating tumorigenesis (Yang et al., 2019). Meanwhile, CircZFR also competitively binds to miR-624-3p, thereby relieving its suppression of WEE1 and promoting the malignant phenotype of HCC cells (Zhang et al., 2024). In addition to its role in oncogenic signaling, CircZFR is also closely associated with chemotherapy resistance in HCC. CircZFR expression is significantly upregulated in DDP-resistant HCC cell lines. CircZFR is highly expressed in cancer-associated fibroblasts (CAFs) and their exosomes, and can be transferred to HCC cells, where it inhibits the STAT3/NF-κB signaling pathway, promoting tumor growth and increasing resistance to DDP (Zhou et al., 2022).

In summary, CircZFR promotes HCC progression by regulating multiple miRNA–target gene axes and is strongly linked to resistance to cisplatin. It plays a multifaceted role in regulating key biological processes such as cell proliferation, migration, EMT, and resistance to chemotherapy. Thus, CircZFR represents not only a prognostic biomarker but also a promising therapeutic target in HCC.

## CircZFR in breast cancer

BC is the most common malignancy among women worldwide, accounting for approximately 11.7% of all new cancer cases and ranking as the second leading cause of cancer-related death in women. Despite advances, early diagnosis and precise treatment of BC remain challenging, highlighting the need for a deeper understanding of its molecular mechanisms to inform more effective prevention and therapeutic strategies.

CircZFR is significantly overexpressed in BC tissues and cell lines. Chen Zhuo et al. discovered that CircZFR functions as a molecular sponge by binding to and inhibiting miR-578, thereby relieving its suppression of the downstream target gene HIF1A.By adsorbing and inhibiting miR-578, it relieves its suppression of the downstream target gene HIF1A, thereby promoting BC cell survival, clonogenic formation, migration, invasion, and glycolysis while inhibiting apoptosis. This study confirms that circZFR drives BC progression by regulating the miR-578/HIF1A signaling axis. Its overexpression is closely associated with malignant phenotypes, demonstrating clinical value as a potential therapeutic target (Chen et al., 2020).

CircZFR is significantly overexpressed in BC and functions as a molecular sponge for miR-578, regulating the miR-578/HIF1A signaling axis. This promotes cell survival, clonogenicity, migration, and invasion, while enhancing glycolysis and suppressing apoptosis. Elevated expression of CircZFR is associated with the malignant phenotype of BC, highlighting its potential as a therapeutic target for further clinical investigation. These findings offer novel insights into the early diagnosis and precision therapy of BC.

## CircZFR in lung cancer

LC is the third most common malignancy worldwide and remains a leading cause of cancer-related mortality. In 2022, LC accounted for 22.0% of all new cancer cases and 28.5% of cancer-related deaths (Wang et al., 2022). Although early-stage LC is associated with a relatively favorable prognosis, most patients are diagnosed at an advanced stage due to the limited effectiveness of early screening, leading to poor overall outcomes. Therefore, advancing our understanding of LC pathogenesis and identifying novel biomarkers or therapeutic targets may offer valuable insights into the early diagnosis and effective treatment of advanced-stage disease (Siegel et al., 2025).

Studies have shown that CircZFR is significantly overexpressed in LC tissues compared to adjacent normal tissues, suggesting its potential as a novel biomarker for LC. CircZFR enhances the expression of Cyclin D1, encoded by the CCND1 gene, by maintaining high levels of H3K4me3 at the CCND1 promoter. This activates the G1–S phase transition in the cell cycle of LC cells and significantly promotes tumor cell proliferation and metastasis (Ren et al., 2020). Zhang et al. further investigated the role of CircZFR in the pathogenesis of non-small cell lung cancer (NSCLC). Mechanistic studies reveal that circZFR sequesters miR-101-3p via sponge-like adsorption, thereby releasing miR-101-3p’s suppression of its downstream target gene CUL4B. This leads to upregulation of CUL4B expression, ultimately promoting proliferation, migration, and invasive capabilities in NSCLC cells. This direct molecular interaction and sequential regulatory pathway have been validated through multiple methods, including luciferase reporter assays and functional recovery experiments. Additionally, CircZFR may be involved in the development of chemotherapy resistance in NSCLC cells. CircZFR downregulates the tumor-suppressive miR-545-3p, thereby impairing the protective role of CBLL1 against DDP-induced damage in NSCLC cells, ultimately promoting NSCLC progression (Li et al., 2020). Furthermore, CircZFR depletion enhances the effects of DDP by reducing colony formation, further inhibiting cell cycle progression and metastasis, and increasing DDP-induced apoptosis and necrosis in NSCLC cells (Li et al., 2020). Additionally, Research by Zhifei Ma et al. also contributes to the investigation of the potential mechanisms of circZFR in energy stress. The researchers found that during the development of LC, particularly lung adenocarcinoma (LUAD), tumor cells upregulate CircZFR to cope with energy deficiency. Elevated CircZFR protects heterogeneous nuclear ribonucleoprotein L-like (HNRNPLL) from ubiquitination and degradation, thereby regulating the alternative splicing of MYO1B. This process activates the AKT–mTOR signaling pathway, modulates oxidative phosphorylation (OXPHOS), and enhances mitochondrial energy production, ultimately increasing energy stress tolerance and supporting tumor cell proliferation, metastasis, and invasion (Ma et al., 2023).

Collectively, these studies indicate that CircZFR plays a critical role in the progression of LC and may represent a promising therapeutic target for its treatment. Additionally, the marked difference in CircZFR expression between LC cells and adjacent normal tissues suggests its potential as a biomarker for early-stage LC, offering promising prospects for early detection and diagnosis.

## CircZFR in cervical cancer

CC is a gynecological malignancy primarily caused by persistent infection with human papillomavirus (HPV). It is often asymptomatic in its early stages. Despite the increasing implementation of HPV vaccination and early screening programs, CC remains the fourth most common cancer among women worldwide. In certain regions, it continues to be a leading cause of infertility and mortality in young women (Canfell et al., 2020; Siegel et al., 2020). CircZFR has been closely linked to the progression of CC. CircZFR activates the Rb–E2F1 signaling pathway in CC cells by increasing phosphorylation of p-Rb at S807 and S608, and acetylation of ac-E2F1 at K117 and K125. These modifications enhance the interaction between Rb and the E2F transactivation domain, thereby promoting G1/S phase transition, cell proliferation, migration, and invasion in cervical squamous cell carcinoma (Zhou et al., 2021). Additionally, CircZFR promotes the assembly and activation of the CDK2/cyclin E1 complex by interacting with the protein chaperone SSBP1. Notably, suppression of SSBP1 expression not only disrupts the assembly of the SSBP1/CDK2/cyclin E1 complex, but also reduces phosphorylation of p-Rb at S807 and S608, as well as acetylation of ac-E2F1 at K117 and K125,however, E2F1 inhibition is difficult to achieve to the same extent, suggesting that SSBP1 may serve as a key mediator of CircZFR-driven CCprogression. Targeting CircZFR via the CircZFR–SSBP1/CDK2/cyclin E1 axis may hold therapeutic potential for the treatment of advanced cervical squamous cell carcinoma. Additionally, because early-stage CC is often asymptomatic, there is an urgent need to improve early diagnostic methods, and CircZFR may serve as a promising biomarker.

## Discussion

CircZFR has been frequently implicated in tumorigenesis and progression (Chen et al., 2024; Jiang et al., 2023). Current research on circZFR primarily focuses on the subtype hsa_circ_0072088. This review systematically summarizes the research progress of circZFR in various malignant tumors, encompassing its biological functions and molecular mechanisms. It clearly demonstrates its crucial regulatory role and specific regulatory mechanisms in tumorigenesis, progression, and therapeutic response (Long et al., 2022; Wei et al., 2018).

Multiple studies indicate that circZFR is overexpressed in various malignant tumors, and its abnormal expression is typically significantly associated with tumor progression, TNM staging, as well as prognosis, survival rates, and quality of life. Thus, circZFR primarily functions as an oncogene (Chen et al., 2024; Wei et al., 2018). Regarding its mechanism of action, circZFR can regulate downstream signaling pathways by acting as a miRNA sponge or directly bind functional proteins to modulate tumor cell proliferation, invasion, metastasis, and other processes. It is also closely associated with the development of cisplatin resistance in HCC and NSCLC. Notably, whether circZFR may exhibit tumor-suppressing functions under specific tumor contexts or microenvironmental conditions remains to be further explored. This knowledge gap limits our understanding of circZFR’s precise function and hinders its translational potential. Its dual role within complex regulatory networks may be significantly influenced by tissue type, molecular context, or tumor microenvironment a particularity warranting in depth investigation (Niu et al., 2025). Therefore, circZFR holds potential as a diagnostic, prognostic, and therapeutic target. In-depth investigation into its mechanisms of action may offer novel insights and directions for cancer therapy (Jiang et al., 2023; Long et al., 2022).

In 2011, Salmena et al. first proposed the competitive endogenous RNA (ceRNA) regulatory mechanism, whereby miRNAs and non-coding RNAs can recognize specific target sites on different RNA molecules, inducing the formation of silencing complexes to inhibit or block downstream signaling pathways. Based on this hypothesis, it is reasonable to infer that circZFR, which is overexpressed in various malignant tumors, competitively binds to miRNAs, reducing the pool of free miRNAs and indirectly affecting the expression of downstream genes, thereby exerting a pro-cancer effect. This conjecture has been validated in multiple studies, where researchers successively identified several signaling pathways closely associated with malignant tumor progression (Chen et al., 2020; Li et al., 2020). For instance, Chen et al. discovered the CircZFR-miR-3127-5p/RTKN2 pathway in CRC, Hong et al. identified the CircZFR-miR-1261/C8orf4 pathway in TC, and the CircZFR-miR-101-3p/CUL4B pathway in NSCLC (Chen et al., 2024; Wei et al., 2018; H. Zhang et al., 2019). Furthermore, the role of circZFR in promoting cisplatin resistance in HCC cells warrants further investigation. Research indicates that circZFR transported into HCC cells reduces their sensitivity to cisplatin by inhibiting the STAT3/NF-κB signaling pathway. Conversely, blocking circZFR transport or inhibiting its synthesis may enhance cisplatin’s cytotoxic effects on HCC cells, potentially offering new therapeutic options for patients with refractory HCC or cisplatin resistance. Research indicates that circZFR transported into HCC cells reduces their sensitivity to cisplatin by inhibiting the STAT3/NF-κB signaling pathway. Conversely, blocking circZFR transport or inhibiting its synthesis may enhance cisplatin’s cytotoxic effects on HCC cells, potentially offering novel therapeutic options for patients with refractory HCC or cisplatin resistance (Li et al., 2020; Zhou et al., 2022). It is worth noting that circZFR does not exert a singular pro-cancer effect across all malignant tumors. In CRC, circZFR dynamically regulates the expression level of downstream FOXO4 by binding to miR-532-3p, thereby promoting apoptosis and inhibiting cell cycle progression, thus exerting a certain anti-cancer effect (Bian et al., 2018). This diametrically opposed effect suggests that transforming circZFR’s “oncogenic advantage” in malignant tumor progression into an “anti-cancer advantage” may offer a novel therapeutic approach. Furthermore, unlike previous reports on the functions of other circRNAs such as circHIPK3 and circSMARCA5, circZFR exhibits unique tissue specificity and functional diversity in certain cancer types. It may participate in the tumor microenvironment by regulating immune responses, promoting angiogenesis, and influencing matrix remodeling (Long et al., 2022; Ma et al., 2023). This suggests its scope may extend beyond the traditional ceRNA model, potentially involving a more complex biological network. In-depth investigation of this network could provide novel approaches for early diagnosis, treatment, and improved prognosis. Additionally, circZFR can directly interact with RNA-binding proteins, participating in gene expression regulation through non-canonical mechanisms involving transcription, translation, and epigenetic modifications. However, to date, no specific, conclusive mechanisms have been reported or confirmed, and further exploration of these mechanisms remains necessary.

Although circZFR has been demonstrated to play a crucial regulatory role in various malignant tumors, research into its mechanisms and clinical value remains limited (Chen et al., 2024; Long et al., 2022). Current studies are predominantly confined to cellular and animal experiments, focusing primarily on mechanism investigation. There is a lack of large-scale, multicenter prospective clinical trials to validate its clinical efficacy and stability. How to translate findings from mechanism research into practical clinical applications remains a significant challenge. Furthermore, while multiple studies have identified circZFR overexpression in malignant tumors, suggesting its association with poor tumor prognosis, no definitive research has yet confirmed that elevated circZFR expression constitutes an independent adverse prognostic factor promoting tumor progression. This uncertainty limits the clinical application and promotion of circZFR. Furthermore, inconsistent methods for circRNA enrichment, quantification, and analysis across studies compromise data comparability and reproducibility, hindering practical application. While circZFR’s closed-loop structure confers high stability, it also increases targeting complexity. Current strategies for targeting circRNA primarily rely on antisense oligonucleotides targeting specific cleavage sites, CRISPR/Cas systems, and small-molecule inhibitors or RNA aptamers. Nevertheless, delivery efficiency, tissue specificity, and potential off-target effects remain major bottlenecks in circRNA-targeted therapies, similarly hindering the clinical translation of circZFR-related research findings (Drula et al., 2023).

In summary, circZFR, as a functionally complex circRNA, plays a key regulatory role in various malignant tumors and holds potential as a diagnostic biomarker and therapeutic target (Jiang et al., 2023). In-depth analysis of its molecular mechanisms and clinical value not only enriches the theoretical framework of tumorigenesis and progression but also provides a crucial research foundation and practical pathway for achieving precision cancer therapy (Bian et al., 2018; Wei et al., 2018). Future research should integrate multi-omics approaches such as single-cell sequencing, spatial transcriptomics, and RNA modification omics. By focusing on sub-type structures, tumor microenvironments, and signaling pathways, we can precisely decipher circZFR’s dynamic roles across different tumor stages and microenvironments. Clarifying its network relationships with key signaling pathways and regulatory factors will enable the reversal of its “oncogenic advantage” into an “anti-cancer advantage,” accelerating the translation of experimental findings into clinical applications.
